# Influence of Factor V Leiden on susceptibility to and outcome from critical illness: a genetic association study

**DOI:** 10.1186/cc8899

**Published:** 2010-03-05

**Authors:** Thomas Benfield, Karen Ejrnæs, Klaus Juul, Christian Østergaard, Jannik Helweg-Larsen, Nina Weis, Lea Munthe-Fog, Gitte Kronborg, Marianne Ring Andersen, Anne Tybjærg-Hansen, Børge G Nordestgaard, Peter Garred

**Affiliations:** 1 Department of Infectious Diseases and Clinical Research Centre, Hvidovre University Hospital, Kettegaard Alle 30, 2650 Hvidovre, Denmark; 2 Faculty of Health Sciences, University of Copenhagen, Blegdamsvej 3, Copenhagen, DK-2200, Denmark; 3 Department of Clinical Microbiology, Hvidovre University Hospital, Kettegaard Alle 30, Hvidovre, DK. 2650, Denmark; 4 Department of Clinical Biochemistry, Herlev University Hospital, Herlev Ringvej 75, Herlev, DK-2730, Denmark; 5 Pediatric Cardiology Section, Department of Pediatrics, Copenhagen University Hospital, Blegdamsvej 9, Copenhagen, DK-2100, Denmark; 6 Department of Clinical Microbiology, Herlev University Hospital, Herlev Ringvej 75, Herlev, DK-2750, Denmark; 7 Department of Infectious Diseases, Copenhagen University Hospital, Blegdamsvej 9, Copenhagen, DK-2100, Denmark; 8 Laboratory of Molecular Medicine, Department of Clinical Immunology, Copenhagen University Hospital, Blegdamsvej 9, Copenhagen, DK-2100, Denmark; 9 Department of Clinical Biochemistry, Copenhagen University Hospital, Blegdamsvej 9, Copenhagen, DK-2100, Denmark; 10 The Copenhagen City Heart Study, Bispebjerg University Hospital, Bispebjerg Bakke 23, Copenhagen, DK-2400, Denmark

## Abstract

**Introduction:**

Disturbance of the pro-coagulatant and anti-coagulant balance is associated with a poor outcome from critical illness. The objective of this study is to determine whether the Factor V Leiden (FVL) mutation is associated with susceptibility to or death from critical illness.

**Methods:**

A genetic association study involving four case cohorts comprising two Gram negative sepsis, one invasive pneumococcal disease and one intensive care unit cohort with a total of 1,249 patients. Controls were derived from a population-based cohort study (N = 8,147). DNA from patients and controls was genotyped for the FVL mutation.

**Results:**

When all patients were investigated together no significant difference in the frequency of FVL mutation was observed compared with controls (odds ratio (OR), 1.03; 95% confidence interval (CI), 0.83 to 1.29). However, when stratified among patients admitted to intensive care (N = 237), susceptibility and the likelihood of long-term death was influenced by the FVL mutation. In adjusted logistic regression analysis, FVL carriers had an increased risk of ICU admission compared to non-carriers (OR 1.62; 95% CI, 1.08 to 2.42). In adjusted Cox regression analysis, FVL carriers were at increased risk of long-term death compared to non-carriers (relative risk 1.78; 95% CI, 1.13 to 2.81). FVL carrier status did not predict either susceptibility to or outcome from Gram negative, *Escherichia coli *or *Streptococcus pneumoniae *sepsis.

**Conclusions:**

Overall, the FVL mutation did not appear to increase the risk of admission due to severe invasive infections. Nevertheless, in the subgroup of patients admitted to intensive care an increased risk and a poorer long-term outcome for individuals with critical illness were observed for FVL mutation carriers.

## Introduction

Critical illness associated with sepsis and the systemic inflammatory response syndrome (SIRS) is an important cause of morbidity and mortality [1-3]. In recent years, a growing number of discoveries have identified the importance of host genetic factors in SIRS and sepsis outcomes [4].

One human genetic factor that may be involved is factor V but its role is controversial. A single non-synonymous amino acid substitution (Arg506Gln) in factor V, the factor V Leiden (FVL) mutation, causes resistance to activated protein C (APC) leading to increased levels of thrombin [5]. The FVL mutation *per se *is associated with an increased risk of thromboembolism [6,7].

Clinical and experimental studies that have investigated the effect of the FVL mutation on sepsis outcomes have come to conflicting conclusions. In one clinical trial, FVL carriers with severe sepsis had a survival benefit compared to non-carriers [8]. Similarly, FVL carriage appeared to be associated with improved short-term survival from the acute respiratory distress syndrome (ARDS) [9]. Findings from one experimental endotoxemia model supported this [8]. Other studies have found either no effect or a detrimental effect of FVL carriage. In an observational cohort, the FVL mutation did not affect survival from severe sepsis [10]. Genetically engineered mouse models of peritonitis and group A streptococcal (GAS) disease showed increased mortality from sepsis and an increased susceptibility to GAS infection [11,12]. In meningococcal disease, FVL carriage was associated with increased morbidity [13] and in sepsis with a four-fold increased relative risk of mortality [14]. In the latter study, susceptibility to different manifestations of infectious diseases was also influenced by FVL carriage.

The aim of the present study was to study the possible association of FVL with critical illness caused by sepsis and SIRS. To this end, we genotyped five cohorts including individuals without infection, individuals with SIRS and individuals with sepsis caused by Gram negative bacteria and *Streptococcus pneumoniae*. The allele and carrier frequencies were analyzed for association with susceptibility to infection and outcome from infection.

## Materials and methods

### Cohorts

#### Control group

All subjects who participated in the 1991 to 1994 Copenhagen City Heart Study (CCHS) were included in the control group if they had not had an infectious disease hospitalization by 31 December 2001 or a case of invasive pneumococcal disease (IPD) by 1 October 2007. Subjects ≥ 20 years old were selected randomly after age stratification from among residents of Copenhagen. Of the 17,180 individuals invited, 10,135 participated, 9,259 provided blood samples, and 9,253 were genotyped for factor V Leiden. A total of 1,106 individuals were excluded because they had been hospitalized at least once with an infectious disease. Thus, a total of 8,147 adults comprised the control group. Ninety-nine percent were of Danish decent [15]. Details of study procedures have been described elsewhere [6]. All subjects provided written, informed consent, and the ethics committee for Copenhagen and Frederiksberg approved the study (Record no. 100.2039/91).

#### Gram negative sepsis cohort 1 (G1)

There were 452 consecutive episodes of Gram negative bacteremia among 427 individuals admitted to Hvidovre Hospital from June 2000 through May 2002. Of these, 319 were a first episode of bacteremia during the study period and had DNA collected. None of the patients were lost to follow-up. Ninety-five percent were of Danish decent. Details of the study are described elsewhere [16]. The study was approved by the Ethics Committee for Copenhagen and Frederiksberg counties (record no. 01-085/2000).

#### Gram negative sepsis cohort 2 (G2)

All patients older than 18 years admitted to Amager Hospital, Bispebjerg Hospital, Frederiksberg Hospital or Hvidovre Hospital in Copenhagen from January 2003 through May 2005 with a positive blood and urine culture yielding *Escherichia coli *were included in the study. A total of 575 consecutive episodes of *E. coli *bacteremia with bacturia were included. None of the patients were lost to follow-up. The study was approved by the Ethics Committee for Copenhagen and Frederiksberg counties (record no. 01-2006-6173).

#### Intensive care unit (ICU) cohort

From February 1998 to July 1999, 272 individuals admitted to the academic, multidisciplinary ICU at Glostrup Hospital, who met the criteria for SIRS, as outlined by Bone et al. [17], were included in the study. Respiratory failure requiring intubation is generally required for ICU admission in Denmark. All individuals in this cohort were intubated and mechanically ventilated. Details are described elsewhere [18]. Informed consent was obtained from all patients or from their close relatives. The study was approved by the local ethics committee for Copenhagen County (record no. KA 96097).

#### Invasive pneumococcal disease (IPD) cohort

Two studies contributed cases to the IPD cohort. In the 1991 to 1994 CCHS, cases (N = 52; 44 with FV genotype) were identified through linkage with the National Streptococci Reference laboratory (NSR), Statens Serum Institut [19]. From 141 adults included in a study of mannan-binding lectin (MBL) genotypes and IPD, 119 had DNA available for FV genotyping [20]. In total, the IPD cohort comprised 163 individuals. Informed consent was obtained from all patients. The study was approved by Ethics Committee for Copenhagen and Frederiksberg Counties (record no. H-KF-01-152/99).

### Factor V genotypes

Genomic DNA was extracted and stored at -20°C. The FVL mutation (Arg506Gln) was identified by one of three methods. Restriction fragment length polymorphism PCR was used for the CCHS control group, G1 and IPD cohort as described [6,21,22]. Light Cycler technology (Roche, Basel, Switzerland) was used to determine the FVL genotype in the G2 cohort as described [23]. The ICU cohort was genotyped with the TaqMan MGB method. In brief, single-nucleotide variants were detected using Taq Man MGB probe assay (C__11975250_10, TaqMan^® ^MGB assay, Applied Biosystems, Foster City, CA, USA) according to the manufacturer's instructions. Four controls were added for every 44 reactions: two allelic controls (one homozygous for the minor allele and one heterozygous; major/minor) and two non-template controls. PCR was performed under the following conditions: 1 × 10 minutes 95°C and 40× (15 sec 92°C, 1 minute 60°C). Results were analyzed on ABI PRISM 7700 Sequence Detection platform using the SDS software v1.9 (Applied Biosystems), and using the allele discrimination plate read function to detect the end-point fluorescence in each well. Genotype results were manually assigned. Sanger-sequencing was applied to two randomly chosen patients on each plate (ABI Prism 3100 Genetic Analyser, Applied Biosystems).

### Statistics

Genotype distributions were tested for Hardy-Weinberg equilibrium by using χ^2 ^tests. Allele, genotype, carrier frequency and demographic features were evaluated by using χ^2 ^or Fisher exact tests, whenever appropriate. We calculated odds ratios (ORs) and 95% confidence intervals (CI) when appropriate. Multiple logistic regression analysis was done to evaluate the association between FVL and susceptibility to infection after adjusting for age and sex.

In survival analysis, heterozygous and homozygous individuals were combined (carriers) and compared with noncarriers. Cox regression analysis examined time to death by using hazard ratios (HR) with 95% CIs. Covariates that were associated with death in univariate analysis were included in the multivariate model. Short-term survival was defined as outcome at Day 30 after admission for infection or critical illness. Long-term outcome was defined survival status at the end of follow up. Statistical analysis was performed with SPSS 17.0 (Statistical Package for Social Sciences, Chicago, IL, USA). We considered two-tailed *P *values of < 0.05 to be statistically significant.

## Results

The genotype distribution did not differ from that predicted by Hardy-Weinberg equilibrium for any of the five cohorts (*P *> 0.2 for all). Characteristics of subjects in each cohort are shown in Table 1. When all patients were investigated together no significant difference in the frequency of FVL mutation was observed compared with controls (OR 1.03; 95% CI, 0.83 to 1.29).

**Table 1:** Characteristics of the five cohorts

	Controls N = 8147	All cases N = 1249	ICU cohort N = 237	G1 cohort N = 315	G2 cohort N = 534	IPD cohort N = 163
Age, yrs Median (IQR)	66 (54 to 76)	74 (61 to 83)*	64 (52 to 73)*	76 (61 to 84)*	78 (68 to 86)*	69 (56 to 78)
Female, %	55.7	55.2	48.5*	54	59.6	52.8

*, *P *< 0.05 compared to control group (Mann-Whitney's or Fisher's exact test). IQR: interquartile range; ICU: intensive care unit; G1: Gram negative sepsis cohort 1; G2: Gram negative sepsis cohort 2; IPD: invasive pneumococcal disease.

### Intensive care unit cohort

From the ICU cohort, 237 individuals (87%) had DNA available for analysis. Individuals excluded because DNA was unavailable were significantly younger (57 vs. 64 years, *P *= 0.006) than individuals included but did not differ with respect to sex or markers of disease severity at baseline. Overall the ICU cohort was slightly younger and had more males than the control cohort (Table 1). Using χ^2 ^statistics to compare allele and genotype frequencies we found that patients admitted to the ICU more often were carriers of the A allele and the GA genotype compared to controls. After adjustment for age and sex, the odds ratios correlating to the A allele and GA genotype with ICU admission remained statistically significant (1.66 (1.11 to 2.4) and 1.62 (1.08 to 2.42), respectively; Table 2).

**Table 2:** Factor V allele and genotype frequencies in individuals with systemic inflammatory response syndromeand sepsis compared to controls

		Controls N = 8147	All cases N = 1249	ICU cohort N = 237	G1 cohort N = 315	G2 cohort N = 534	IPD cohort N = 163
Number and frequency of the FV alleles (%)	G A	15648 (96.0) 646 (4.0)	2396 (95.9) 102 (4.1)	446 (94.1) 28 (5.9)	607 (96.3) 23 (3.7)	1025 (95.9) 43 (4.0)	318 (97.5) 8 (2.5)
Allele *P *value		--	Chi Sq = 0.0517; df = 1; *P *= 0.820	Chi.Sq = 4.0156; df = 1; *P *= 0.0451	Chi.Sq = 0.0856; df = 1; *P *= 0.7699	Chi.Sq = 0.004; df = 1; *P *= 0.984	Chi.Sq = 1.5506; df = 1; *P *= 0.2131
OR (95% CI)		--	1.03 (0.83 to 1.28)	1.52 (1.03 to 2.25)	0.97 (0.63 to 1.49)	0.92 (0.65 to 1.30)	0.63 (0.31 to 1.30)
Adj. OR (95% CI)*		--	1.02 (0.82 to 1.29)	1.66 (1.11 to 2.49)	0.97 (0.63 to 1.50)	0.92 (0.65 to 1.30)	0.63 (0.31 to 1.30)
Allelic *P *value**		--	0.871	0.014	0.897	0.626	0.216
Number and frequency of the FV genotype (%)	GG	7518 (92.3)	1150 (92.1)	209 (88.2)	292 (92.7)	494 (92.5)	155 (95.1)
	GA	612 (7.5)	96 (7.7)	28 (11.8)	23 (7.3)	37 (6.9)	8 (4.9)
	AA	17 (0.2)	3 (0.2)	0	0	3 (0.6)	0
Genotypic *P *value		--	0.952	0.04	0.71	0.3	0.23
OR (95% CI)*** Carrier vs. non-carrier		--	1.03 (0.83 to 1.28)	1.60 (1.07 to 2.39)	0.94 (0.61 to 1.45)	0.97 (0.69 to 1.35)	0.62 (0.30 to 1.26)
Adj. OR (95% CI)* Carrier vs. non-carrier		--	1.03 (0.83 to 1.29)	1.62 (1.08 to 2.42)	0.95 (0.61 to 1.46)	0.97 (0.69 to 1.36)	0.62 (0.30 to 1.27)
Genotypic *P *value**		--	0.791	0.019	0.799	0.847	0.189

*: Adjusted for age and sex. **: adjusted analysis. ***: logistic regression analysis of carriers (GA or AA) vs. non-carriers. CI, confidence interval; OR, Odds ratio. ICU: intensive care unit; G1: Gram negative sepsis cohort 1; G2: Gram negative sepsis cohort 2; IPD: invasive pneumococcal disease.

**Figure 1:**
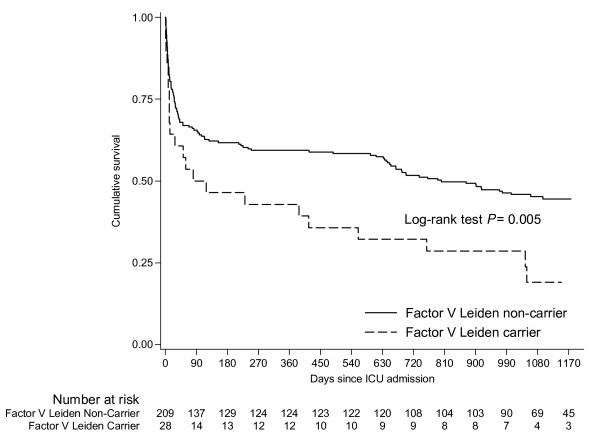
**Survival after admission to intensive care among carriers and non-carriers of the factor V Leiden mutation**.

Short-term mortality rates were higher among carriers admitted to the ICU than non-carriers but the difference was not statistically significantly different (Table 3). However, long-term survival was affected by FVL carrier status. During a median of 698 (IQR: 24 to 1117) days of follow up, 23 of 28 (82.1%) carriers compared to 115 of 209 (55%) non-carriers died (log-rank test, *P *= 0.005, Figure 1). In multivariate analysis, adjusting for factors that were associated with outcome in univariate analysis, FVL carrier status, age and SAPS II score at baseline were associated with long-term outcome (Table 4).

**Table 3:** Mortality rates associated with Factor V carrier status in individuals with critical illness

	Mortality		
	**30-day**	**90-day**	**Overall**

**ICU cohort**			
Non-carrier	55/209 (26.3)	72/209 (34.4)	115/209 (55)
Carrier	11/28 (39.3)	14/28 (50)	23/28 (82.1)*
**G1 cohort**			
Non-carrier	40/292 (13.7)	70/294 (24)	90/292 (30.8)
Carrier	5/23 (21.7)	8/23 (34.8)	8/23 (34.8)
**G2 cohort**			
Non-carrier	80/494 (16.2)	113/494 (22.9)	275/494 (56.0)
Carrier	10/40 (25.0)	11/40 (27.5)	21/40 (52.5)
**IPD cohort**			
Non-carrier	24/155 (15.5)	NA	NA
Carrier	1/8 (12.5)		

*: Fisher's exact test *P *= 0.007. ICU: intensive care unit; G1: Gram negative sepsis cohort 1; G2: Gram negative sepsis cohort 2; IPD: invasive pneumococcal disease.

**Table 4:** Multivariate analysis of factors associated with long-term mortality after ICU admission

	Univariate analysis HR (95%)	Multivariate analysis HR (95%)	*P *value
**FVL**			
Non-carrier	1.0	1.0	
Carrier	1.88 (1.20 to 2.95)	1.78 (1.13 to 2.81)	0.013
**Age**			
(per year increment)	1.04 (1.03 to 1.05)	1.02 (1.01 to 1.03)	0.002
**SAPS II**			
(per point increment)	1.05 (1.04 to 1.06)	1.03 (1.02 to 1.05)	0.0001

FVL, Factor V Leiden; HR, hazard ratio; SAPS II, Simplified acute physiology score II.

### Other cohorts

FVL carrier status did not predict either susceptibility to or outcome from Gram negative sepsis, *E. coli *sepsis, *S. pneumonia *sepsis or the combined case cohort (Tables 2 and 3).

## Discussion

Our findings suggest that a genetic disposition to coagulation, FVL, may be associated with susceptibility to and outcome from critical illness.

The present study and its findings add to a growing number of studies in apparent disagreement. However, the published studies are not immediately comparable. The animal models used transgenic mice but otherwise differed in their approach and in their findings. Kerlin et al. used injectable lipopolysaccharide (LPS) from *E. coli *to elicit sepsis and showed that FVL was associated with improved survival [8]. Sun et al. induced sepsis through subcutaneous injection of GAS and showed a marked relationship between the FV deficiency and increased mortality [12]. Brüggemann et al. used a cecal ligation and puncture method to induce sepsis and showed that FVL was disadvantageous for short-term survival [11]. The five clinical studies also differ significantly. Two showed a beneficial effect, two a detrimental effect of FVL carriage, and one showed no effect of FVL carriage on outcome from severe sepsis [8-10,13,14]. Kerlin et al. applied a post hoc analysis to a randomized clinical trial (RCT) of adult patients with severe sepsis in whom FVL was beneficial for short-term survival. Although, this study is closest to our ICU population, a RCT represents a selected population eligible to study specified inclusion criteria, and, thus, may be subject to selection bias [8]. Adamzik et al. included individuals admitted to intensive care who had ARDS [9]. FVL was associated with improved short-term survival in patients with ARDS but, although the majority also had an infection (pneumonia or sepsis), ARDS represents a distinct population with a different disease entity. Kondaveeti et al. studied children who had meningococcal disease and showed an association between FVL and disease severity but not to outcome [13]. The study size, however, had limited statistical power to detect a difference in outcome because the mortality rate was low. Our own study relied on hospital discharge records and included a limited number of individuals hospitalized with sepsis [14]. Nevertheless, the detrimental effect of the FVL mutation was striking. Thus, the published studies are based on heterogeneous patient populations and the differences between them emphasize the need for more research in this area.

The increased risk of critical illness associated with FVL carriage is not immediately explained. FVL leads to greater risk of thromboembolism and confers resistance to APC [6]. Treatment with APC has been shown to improve survival from severe sepsis [24]. The mechanisms responsible for the benefit of APC are unknown but believed to derive from APC's anti-inflammatory and anti-coagulation properties. We speculate that individuals with FVL have reduced intrinsic anti-inflammatory potential leading to vasodilation and suffer from disturbances of the pro- and anti-coagulant balance leading to microthrombolism. Vasodilation and microthrombolism in combination likely impair blood circulation and accentuate the effects of sepsis.

FVL affected long-term survival from critical illness. It is unclear what may explain this correlation. One explanation may be that critical illness altered the comorbid risk profile of patients after discharge. Smeeth et al. have shown that acute lower respiratory tract infections and urinary tract infections are associated with a transient increase in the risk of a vascular event (myocardial infarction, stroke and venous thromboembolism) [25,26]. The mechanisms by which acute inflammation may affect the risk of vascular events are uncertain but may include endothelial dysfunction. Since FVL is associated with an increased risk of thromboembolism per se it is possible that sepsis and SIRS induced inflammation further increased the risk of a vascular event after an admission to intensive care for critical illness. Unfortunately, we are unable to investigate this in more detail because our study does not include information on specific causes of death after discharge from hospital. Future studies are warranted to further investigate this association.

Our study has several limitations. Individuals in the case cohorts were either younger or older than individuals in the control cohort. This may influence the estimates of disease susceptibility because age is one of the most important risk factors for acquisition of infectious disease. However, the effects of Factor V Leiden carrier status did not change significantly when age was included or omitted from the logistic regression analysis.

Ethnicity was unknown in three of the five cohorts. Population stratification may lead to findings that are due to the underlying structure of the population and not the genetic variation being studied. However, we find it unlikely that population stratification has affected our results. In the two cohorts with known ethnic background 99% and 95% of individuals were of Danish decent. The remaining three cohorts were recruited from similar populations in the same geographical area as the cohorts with known ethnic backgrounds.

The G1 and ICU cohorts were established prior to the approval of activated protein C (APC) for treatment of severe sepsis. However, cohort G2 and, in part, the IPD cohort were established after 2002. In theory, use of APC could modulate the effect of the Factor V Leiden mutation. Information on APC use in these two cohorts was unavailable but a very limited number of individuals in all of Denmark received APC during the study period (the average number of APC treatments in Denmark between 2004 and 2008 were 20 per year). Thus, it appears unlikely that use of APC affected our results. The sample size was small to moderate for the four disease cohorts. Consequently, the study may have had insufficient statistical power to detect small and moderate associations between FVL carrier status and disease or disease outcome.

## Conclusions

Overall, the FVL mutation did not appear to increase the risk of admission due to severe invasive infections and was not associated with overall outcome. However, the present study suggests that the FVL mutation may increase the susceptibility to critical illness and may confer a poor long-term outcome of critical illness.

## Key messages

• The FVL mutation was significantly associated with an increased risk of admission to intensive care.

• The FVL mutation was significantly associated with an increased risk of long-term mortality after admission to intensive care.

• The FVL mutation was not associated with an increased risk of severe invasive infections.

## Abbreviations

APC: activated protein C; ARDS: adult respiratory distress syndrome; CCHS: Copenhagen City Heart Study; CI: confidence interval; DNA: dioxynucleotide acid; FVL: factor V Leiden; G1: Gram negative sepsis cohort 1; G2: Gram negative sepsis cohort 2; GAS: group A streptococcus; HR: hazard ratio; ICU: intensive care unit; IPD: invasive pneumococcal disease; LPS: lipopolysaccharide; OR: odds ratio; RCT: randomized controlled trial; SAPS: sepsis acute physiology score; SIRS: systemic inflammatory response syndrome.

## Competing interests

The authors declare that they have no competing interests.

## Authors' contributions

TB conceived the collaborative study. JHL, CØ and TB designed and collected data and samples for the Gram negative sepsis cohort 1. KE and TB designed and collected data and samples for the Gram negative sepsis cohort 2. NW and GK designed and collected data and samples for the invasive pneumococcal disease cohort. ATH and BGN designed and collected data and samples for the Copenhagen City Heart Study. PG designed and collected data and samples for the intensive care unit cohort. KJ, LMF and MRA performed factor V genotyping. TB and PG drafted the first version. All authors read, revised and approved the final version.
